# Feature Detection Techniques for Preprocessing Proteomic Data

**DOI:** 10.1155/2010/896718

**Published:** 2010-05-05

**Authors:** Kimberly F. Sellers, Jeffrey C. Miecznikowski

**Affiliations:** ^1^Department of Mathematics and Statistics, Georgetown University, Washington, DC 20057, USA; ^2^Department of Biostatistics, University at Buffalo, Buffalo, NY 14214, USA; ^3^Department of Biostatistics, Roswell Park Cancer Institute, Buffalo, NY 14263, USA

## Abstract

Numerous gel-based and nongel-based technologies are used to detect protein changes potentially associated with disease. The raw data, however, are abundant with technical and structural complexities, making statistical analysis a difficult task. Low-level analysis issues (including normalization, background correction, gel and/or spectral alignment, feature detection, and image registration) are substantial problems that need to be addressed, because any large-level data analyses are contingent on appropriate and statistically sound low-level procedures. Feature detection approaches are particularly interesting due to the increased computational speed associated with subsequent calculations. Such summary data corresponding to image features provide a significant reduction in overall data size and structure while retaining key information. In this paper, we focus on recent advances in feature detection as a tool for preprocessing proteomic data. This work highlights existing and newly developed feature detection algorithms for proteomic datasets, particularly relating to time-of-flight mass spectrometry, and two-dimensional gel electrophoresis. Note, however, that the associated data structures (i.e., spectral data, and images containing spots) used as input for these methods are obtained via all gel-based and nongel-based methods discussed in this manuscript, and thus the discussed methods are likewise applicable.

## 1. Introduction

 One of the major goals for scientists is to identify biomarkers for patients, thus ultimately providing them with personalized medicine. Personalized medicine provides a patient-specific means by which to target one's disposition to a disease or condition. Recent developments in this area include molecular profiling technologies which may include metabolomic analysis, genomic expression analysis, and proteomic profiling. Specifically, within proteomic profiling, there are several different techniques used to isolate and quantify the proteins within a subject's proteome. The raw data, however, are abundant with technical and structural complexities, making statistical analysis a difficult task. “Preprocessing” (including normalization, background correction, gel and/or spectral alignment, feature detection, and image registration) is therefore often required to account for the systematic biases present in the technology and to reduce the noise in the data. Feature detection (i.e., the detection and quantification of data features, such as peaks in spectral data, or spots in two-dimensional images) is a particularly important component of low-level analysis, because it works to reduce data size and ease subsequent computations.

Feature detection falls under the general subject of mathematical morphology (MM), which began in the 1960s and encompasses methods from statistics, machine learning, topology, set theory, and computer science [1–4]. MM is the science of analyzing and processing geometric structures (e.g., local maxima) in digital images. Examples of common MM functions include opening, closing, thinning, binning, thresholding, and watershed techniques. A key component in MM is the structuring element, that is, the shape used to interrogate the image. In digital images, the structuring element scans the image and alters the pixels in the window content using basic operators. The goal of processing images with MM methods can be to preserve the global features of the image, preserve large smooth objects in an image, denoise images, and detect objects within an image. Situations where MM methods are employed for detection include pedestrian detection [5], tumor mass detection [6], and facial feature detection [7, 8].

### 1.1. Outline of the Paper

This manuscript outlines feature detection methods used via data preprocessing, specifically to detect and quantify the data associated with peptides (or proteins) in various technologies, particularly stemming from gel electrophoresis or mass spectrometry. Section 2 provides background regarding proteomic data analysis. Section 3 explains the general importance of low-level analysis procedures to be performed on the raw data. With the focus for this manuscript being on feature detection, Section 4 discusses proposed approaches for time-of-flight mass spectrometry data, while Section 5 discusses recent work with regard to two-dimensional (2D) gel data. Section 6 concludes the paper with discussion.

## 2. Proteomic Data Analysis

 Proteomics is the study of the proteome, that is, the entire complement of proteins expressed by a genome or organism. From a developmental standpoint, high throughput analysis in the realm of science began with gene expression microarrays [9, 10]. Following the advancements in microarrays, researchers began to develop high-throughput techniques to analyze the proteome.

There are strong similarities between microarray and proteomic data analysis. The overarching biological research goals are similar, namely, to detect statistically significant differential expression (with regard to genes for microarrays, and with regard to proteins in proteomic data) between samples in different treatment groups. Further, there are analogous technological ideas and image processing techniques used to produce the image data. There exist, however, several significant differences that make preprocessing proteomic data and subsequent proteomic data analysis complex. Biologically, a major difference between a genome and proteome is that the genome can be characterized by the sum of sequences of genomic bases, while the proteome requires knowledge of the structure of the proteins and the functional interaction between the proteins. The primary technical difference between these approaches is the means by which the data are provided. While spots from microarray images are arranged in a systematic matrix fashion, protein spots in a gel image or peaks in protein spectra can be more variable with regard to their location, given the procedure that is used to separate proteins. As well, there is a poor correlation between protein and mRNA abundance and, while both methods address the question of differential expression, only proteomic data analysis can also address differential modification (i.e., where the protein is present in both treatment groups, yet its makeup is slightly altered via methylation or phosphorylation).

A nice overview of differential proteomic approaches is provided in [11], with specific emphasis on mass spectrometry approaches and challenges discussed in [12]. In this paper, we examine the most common approaches used to analyze protein abundance, namely, two-dimensional gel electrophoresis (2-DE) and difference gel electrophoresis (DIGE), and time-of-flight mass spectrometry (TOF-MS); tandem mass spectrometry (MS/MS) is also growing in prominence as a means for studying protein differentiation and modification. Sections 2.1, 2.2, and 2.3, respectively, provide further details surrounding these techniques. Meanwhile, nongel-based alternative methods exist for quantitative protein analysis and also make use of MS or MS/MS for feature detection and quantification. We discuss some of these approaches in Section 2.4. Table 1 summarizes method comparisons.

### 2.1. Two-Dimensional Gel Electrophoresis

**Figure 1:**
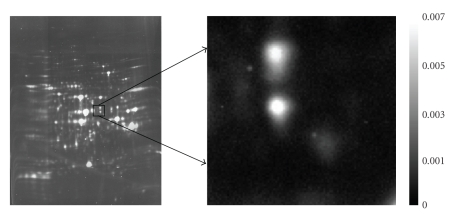
*2-DE Image*: Example of a two-dimensional gel electrophoresis image associated with a particular cyanine dye and light source. Various sources of noise can exist in this image, including general background noise, dust, streaks, and so forth. Further, issues such as low-lying spots and overlapping spots can make spot detection and quantification difficult.

Analysis of quantitative changes in a specific proteome (i.e., complement of proteins expressed in a particular tissue or cell at a given time) is commonly carried out using two-dimensional gel electrophoresis (2-DE). O'Farrell [13] introduces two-dimensional polyacrylamide gel electrophoresis (2D-PAGE), where protein samples are respectively dyed with a cyanine dye (e.g., Cy2, Cy3, or Cy5) and separated in two directions: along the Cartesian *x*-axis by their isoelectric point (pI) via isoelectric focusing, and along the Cartesian *y*-axis by their molecular weight via sodium dodecyl sulphate polyacrylamide gel electrophoresis (SDS-PAGE). The 2D-PAGE technique can be very sensitive to experimental conditions such as laboratory humidity, voltage fluctuations, and gel matrix irregularities. Ünlü et al. [14] suggest an alternative design (namely, the two-dimensional difference gel electrophoresis, or 2D-DIGE approach) to combat some of the inherent data variability that exists in the 2D-PAGE method. Here, after the respective samples are labeled with a particular dye, the samples are then mixed together to create one composite sample where the proteins are, in turn, separated in both directions as described above. In either case, the associated gel(s) is subsequently imaged via a charged-couple device (CCD) camera or a variable mode scanner to produce the raw image data, where the proteins appear as spots; see, for example, Figure 1. These images are then analyzed using an image analysis software tool (e.g., ImageMaster, PDQuest).

2-DE methods such as 2D-PAGE and 2D-DIGE are popular techniques for protein separation because they allow researchers to characterize quantitative protein changes on a large scale. Thus, 2-DE is frequently used as an initial screening procedure whereby results obtained generate new/subsequent hypotheses and determine the direction of ensuing studies. These technologies revolutionized the field of proteomics in their ability to detect protein differences via spot detection and quantification, either with respect to protein expression or modification. Further, they are attractive because of their resolving power, sensitivity, and the low equipment cost. 2-DE analyses, however, require personnel with significant wet laboratory expertise and can be time-consuming, thus potentially limiting the sample size for gels. Furthermore, in some cases (e.g., aging studies, chronic drug treatment, screening for biomarker), replication of the study may be prohibitive. Heterogeneities in different gels, the electric fields, pH gradients, thermal fluctuations, and so forth are all factors that make reproducible spot matching between gels a difficult task. As well, scientists are interested in better tools that allow for a completely automated approach to detect protein changes, particularly in low-abundance proteins. These factors not only make it critically important to correctly analyze the 2-DE results, but also to maximize the information obtained from an experiment.

### 2.2. Mass Spectrometry

**Figure 2:**
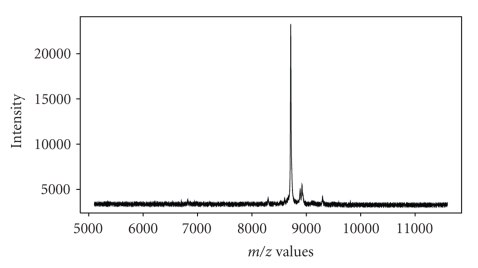
*Mass Spectrometry:* The spectrum contains various kinds of noise that must be addressed via low-level analysis techniques. The focus of this paper addresses peak detection and quantification from such spectra.

Mass spectrometry is an analytic tool used to identify proteins, where the associated instrument (a mass spectrometer) measures the masses of molecules converted into ions via the mass-to-charge (*m*/*z*) ratio. This technology can be used to profile protein markers from tissue or bodily fluids, such as serum or plasma in order to compare biological samples from different patients or different conditions. Matrix assisted laser desorption and ionization—time of flight (MALDI-TOF) is a popular tool used by scientists, where a metal plate with the matrix containing the sample is placed into a vacuum chamber that is excited by a laser, causing the protein molecules to travel (or “fly”) through the tube until they strike a detector that records the time-of-flight for the various proteins under study; surface enhanced laser desorption and ionization—time of flight (SELDI-TOF) is an analog of MALDI-TOF. The interested reader is referred to [15] for discussion regarding the experimental design that creates the data, and elaboration on the MALDI and SELDI constructs. The resulting data are spectral functions containing the *m/z* ratio and associated intensity, where the peaks in the spectral plots correspond to proteins (or peptides) present in the sample; see Figure 2 for an example of a MALDI spectrum. The appeal of mass spectrometry lies in its ability to produce high-resolution measurements with reasonable reproducibility. These procedures generate large amounts of spectral data and can detect protein differential expression and modification in different treatment groups. Noisy data, however, can lead to a high rate of false positive peak identification. This is a significant issue when working to establish an unbiased, automated approach to detect protein changes, particularly in low-abundance proteins.

### 2.3. Tandem Mass Spectrometry

Tandem MS (MS/MS) is an extension of the MS procedure that allows for further fragmentation of protein mixtures. The setup for such a procedure can be physical where two mass spectrometers are assembled in tandem, or the machine may have the ability to store the ions of interest to run the subsequent separation. As a result, the second arrangement allows for continued subsequent operations to be performed. There are various experiments that warrant the use of MS/MS, including product-ion scans, precursor-ion scans, constant neutral-loss scans, and selected reaction monitoring. Product-ion scans determine the product ions that result from decomposing the protein mixture. This experiment is the most common MS/MS experiment [16]. The precursor-ion scan can be thought of as solving the inverse equation, namely, determining the original mixture that could produce the specified product ions. This is useful for determining the makeup of a protein mixture. Constant neutral-loss scans searches for spectra pairs that differ by a constant. This serves to help identify the characteristic mass associated with a protein mixture. Selected reaction monitoring focuses on a preselected mass to identify the makeup of a protein mixture via the use of the associated product or precursor masses. Just as there are various uses for the MS/MS technology, there exist a wide variety of tandem-mass spectrometers, including reverse-geometry MS, triple quadrupole MS, trapped-ion MS, and MALDI-TOF MS/MS; see [16] for details.

MS/MS is valued by scientists for its ability to detect compounds in mixtures. In particular, MS/MS improves the detection limits for some compounds, and improves the signal-to-noise ratio relative to MS. On the other hand, however, the total ion current associated with an MS/MS spectrum is decreased compared to that from a MS spectrum. Further, various ion activation methods affect the efficiency, reproducibility, and feature detection of the associated mass spectra. For a detailed description of the MS/MS procedure and associated technologies, see [16] or [17].

### 2.4. NonGel-Based Methods

Non-gel based methods exist as an alternative for analyzing highly complex protein samples, for example, Multidimensional Protein Identification Technology (MudPIT), isobaric Tag for Relative and Absolute Quantitation (iTRAQ) and Isotope Coded Affinity Tags (ICAT). All of these technologies incorporate the use of MS or tandem MS (MS/MS) to analyze such mixtures.

Multidimensional protein identification technology (MudPIT) analyzes proteomic data by first separating peptides via two-dimensional liquid chromatography, and then detecting protein information using a tandem mass spectrometer. Strengths of this methodology include the orthogonality of the chromatographic phases in the separation process, and its robust representation of separated proteins from complex peptides. Thus, MudPIT is used for a wide range of proteomics experiments, from protein identification and protein cataloging, to quantitative analysis of protein expression. See [18] for an overview of this technology.

Isotope-coded affinity tag (ICAT) is a gel-free, LC-based method for analyzing proteomic data that obtains accurate measurements of protein change, and can analyze sufficient amounts of low-abundance proteins. In the ICAT method, two samples are respectively labeled with either a heavy (i.e., with isotope) or light (without isotope) reagent. The samples are then mixed together and run through an MS or MS/MS machine. The interested reader is referred to [19, 20] for details regarding the ICAT procedure. While this technology provides an accurate measure of relative quantification, it has its share of drawbacks as well. Proteins with little to no cysteine residue are not detected, information can be lost regarding posttranslational modification, and interpreting MS/MS spectra can be difficult because of the addition of the biotin group [20]. Nonetheless, ICAT is a commonly practiced method for analyzing proteomics data.

Isobaric Tag for Relative and Absolute Quantitation (iTRAQ) is a nongel-based alternative to the ICAT method for identifying and quantifying proteins from different samples, having the ability to perform relative or absolute quantification in four or eight phenotypes [21]. The samples are pooled together, and analyzed via MS/MS. Wu et al. [20] found that iTRAQ was more sensitive than DIGE and ICAT with regard to quantitation, but also more prone to errors when performing ion isolation. Gan et al. [21] argue for the use of replicate and pooling samples in iTRAQ studies. By decomposing the overall variation in iTRAQ experiments as either technical or biological, they find that the biological variation outweighs the technical variation in the data, and propose including at least one biological replicate in any iTRAQ experiment.

## 3. Low-Level Analysis

 Various types of noise in the data make protein identification and quantification a difficult problem. Several solutions have been proposed to resolve these issues, yet they remain open problems because of substantial limitations associated with these approaches. Similar to the methods used to analyze gene expression microarrays, the general steps in preprocessing proteomic data include outlier detection, baseline or background subtraction, signal distribution normalization, protein (or peptide) alignment, feature (i.e., peak or spot) detection and quantification, and biomarker evaluation. Concerns regarding these procedures are significant because all subsequent analyses relating to the proteomics data are contingent on these first steps being performed appropriately and optimally. The implications from different pre-processing pipelines are outlined in [22]. Thus, the goal in preprocessing proteomic data is to create an unbiased, reproducible, and automated approach toward identifying differentially expressed and modified proteins, via either spot or peak information differences.

Many of these low level analysis methods are directly integrated into the software that accompanies the mass spectrometer or gel imaging scanner. For example, the DeCyder 2D Differential Analysis software and ImageMaster 2D softwares (all available through GE Healthcare) are often purchased in combination with the 2D gel scanners. Similarly, other commercial softwares available for gel image analysis include PDQuest (Bio-Rad Laboratories), Progenesis SameSpots V3.0 (Nonlinear Dynamics), and Dymension 3 (Syngene). In [23, 24], these softwares are analyzed and compared on several levels including consistency, spot matching accuracy, and spot quantitation. Other softwares and preprocessing methods such as Z3 and Melanie are analyzed and compared in [25–27]. Note, all softwares require user intervention to set parameters and filter settings in order to obtain the optimal preprocessing of the gel image data; this limits the ability for an automated procedure using existing methods. Meanwhile, there are several softwares for preprocessing MS data that are also generally combined with the associated technology. The preprocessing methods in these softwares are often specific to a particular MS structure with algorithms that differ greatly in complexity. Recently, many of the MS preprocessing algorithms have become available to the statistics community through the Bioconductor open source software of *R* libraries [28, 29]. For example, the Bioconductor packages “MassSpecWavelet,” “xcms,” “flagme”, and “TargetSearch” all offer various methods to compare and analyze MS-based datasets [30–33].

While the nongel procedures differ in their protocol, the common denominator with all of these methods lies in their subsequent analysis via MS (or MS/MS). Particularly for ICAT and iTRAQ, they differ only in the number of labeling reagents used, and the distance between and within groups (i.e., peak pairs or groups, depending on the use of the ICAT or iTRAQ method, resp.). Low-lying peaks, however, still remain a problem in that (e.g., with ICAT data) it hinders identification of peak pairs. For both ICAT and iTRAQ data acquired via LC-MS, “different compounds may dilute through the LC column at different speeds” [34], thus hindering the ability to identify peak pairs/groups. This further emphasizes the need for accurate and precise peak/feature detection methods for data stemming from MS and MS/MS technologies.

## 4. Recent Peak Detection in Mass Spectrometry

Proposed procedures for feature (peak) detection in MS data range greatly in algorithm complexity. Although, parsimonious methods should be favored, different variations of the MS technology (e.g., SELDI-MS and MALDI-MS) can require more complex methods to account for systematic biases.

Methods described in [35, 36] take the maximum value within the *k*th nearest neighbors to determine the location of a peak. Yasui et al. [35] apply this approach to preprocess the raw data into local peak/nonpeak binary data. In order to diminish the number of false positives that arose from their choice of *k* = 20, they further define a peak as having an intensity value larger than the average intensity level over a “broad neighborhood” as defined via the super-smoother method with five percent of all data points as the associated smoothing window. Fushiki et al. [36] instead use *k* = 10 after considerable data preprocessing (including baseline correction, averaging the spectra, and spectral alignment via peak matching). Their choice for a smaller *k* better aids in their ability to select peaks that are detected across spectra. Fushiki et al. approach the problem in this manner, because a peak detected in only one spectrum could arguably represent noise, while common peaks across patients may infer the existence of a true biomarker of interest.

Coombes et al. [37] establish a simple peak finding (SPF) algorithm for peak detection in one mass spectrum, where they use a change in slope (via first differences) to detect peaks in SELDI-TOF data. The median absolute value of the first differences is then used to determine the amount of noise in the data, and serves as a threshold for determining what peaks appear to be small enough that they actually represent noise as opposed to true signal. Nearby peaks that fall within a nearness threshold are combined to represent one peak, and the associated peak locations are redefined as the nearest local minima surrounding the local maximum. Finally, the upward and downward slopes are computed for all peaks to quantify peak size and remove small peaks that appear to represent noise. While the SPF algorithm can identify peak location, Coombes et al. [37] warn against solely using this algorithm for peak quantitation as the resulting peak intensity values are not baseline-corrected; however it is sufficient for identifying peak locations. To address peak detection in multiple spectra, they also build from the SPF algorithm to define a simultaneous peak detection and baseline correction (SPDBC) algorithm. The SPDBC algorithm, however does not necessarily identify the same peaks across all spectra. Further, while both algorithms adjust for noise, it still potentially overestimates the number of real peaks in a spectrum depending on the user's determination of certain parameter settings. See [37] for details regarding the SPF and SPDBC algorithms.

Coombes et al. [38] and Morris et al. [39] advocate using an undecimated discrete wavelet transform (UDWT) with hard thresholding to perform peak detection. The UDWT can efficiently separate the signal from noise in the wavelet domain. Coombes et al. [38] perform the UDWT with hard thresholding—transform the data from the time domain to the wavelet domain, set all resulting wavelet coefficients equal to zero that are less than some predetermined threshold, and then transform back into the time domain. This preprocessed data is then baseline-corrected and run through the SPF algorithm described in [37] to locate all peaks in the spectrum. This way, all local maxima are detected. Morris et al. [39] also use a UDWT to perform peak detection but via the mean spectrum. Adapting the algorithm of [38], Morris et al. [39] compute the mean spectrum over all calibrated raw spectra, and apply the essence of the algorithm in [38] to the mean spectrum to denoise, baseline correct, and find peaks. Performing peak detection on the mean spectrum implies increased sensitivity, particularly with regard to low-lying peaks. This algorithm also detects and quantifies peaks without the need for peak-matching algorithms to be applied across samples, because this is addressed inherently through the use of the mean spectrum.

Du et al. [30] create a one-dimensional feature detection algorithm based on the one-dimensional continuous wavelet transform (1D-CWT) to detect peaks in mass spectrometry data. In [30], the 1D-CWT is applied to the raw spectral data thus moving from the time to wavelet domain (using the Mexican Hat wavelet as the mother wavelet), and CWT coefficients are obtained associated with corresponding scales, thus forming a coefficient matrix where, for each scale level, the associated CWT coefficients are maximized at the peak center. This matrix is then visualized via a false color image, matching the ridges in the image to the peaks in the mass spectrum to provide information on how the associated coefficients change across scales. The false-color image ridges correlate well with the spectral peaks, thus providing an alternative approach and visualization tool toward peak detection. The appeal of this approach is its ability to reduce the false positive rate with regard to peak detection and also its lack of dependence on any previous or subsequent preprocessing steps, thus improving the robustness of the results. Assuming a slow-changing, locally monotonic baseline in the spectrum allows for the 1D-CWT to be applied directly to the raw spectrum, and thus there is no need for additional preprocessing to be applied.

Statistical technologies have also been developed for use with MS/MS data or other non-gel-based methods described in Section 2.4, for example, proICAT, SEQUEST, INTERACT, or Hardklör [40]. See von Haller et al. [41], Wu et al. [34], and Hoopmann et al. [40] for details on these respective approaches.

## 5. Recent Spot Detection in Two-Dimensional Gel Electrophoresis

A variety of low-level analysis algorithms exist to summarize information from 2-DE data. Below we focus on three recent algorithms that incorporate or focus on spot detection in gel images.

Srinark et al. [42] have an elaborate, seven-step algorithm for feature detection, including region segmentation, region filtering, spot extraction, centroid estimation, spot merging or splitting, spot filtering, and centroid reestimation. Using the watershed algorithm for initial segmentation, the regions are filtered to focus on regions of reasonable size or variability. The authors then apply *k*-means clustering to each region to differentiate foreground from background pixels, and apply morphological closing to remove noise. The authors then determine an initial spot center estimate for each region, and then reevaluate the initially determined spots via spot splitting and merging to address oversegmentation caused by the watershed procedure. Finally, the algorithm performs another spot filtration procedure to remove features (e.g., dust) from future analysis, and spot centers are reestimated via a 2D Gaussian function. The use of the 2D Gaussian for spot center estimation can be disputed, given that it has long disputed that protein spots are not accurately modeled by a Gaussian distribution. While the algorithm is apparently robust when applied to geometrically distorted simulated images, it has difficulties when applied to real gels, due to difficulty in handling images with differing illuminations, noise, and irregular spot structures; see [42] for details.

Langella and Zivy [43] have established an interesting algorithm that uses image topography to determine spot location and size. In this algorithm, one envisions beads placed at each pixel location within the image, and tracks each bead's progression in the direction of maximal positive slope toward an associated spot's maximum. The associated computer code, available at http://moulon.inra.fr/beads/beads.html, supplies the final image illustrating spot boundaries, along with intermediate grayscale images, including the paths of maximal slope for each respective bead associated with pixel locations in the original image (DIRECTIONS), the number of beads that arrive at each respective location (BEADS), the number of beads that travel through respective pixel locations (PATHS), and possible spot center locations (SELECT). Further, the PROBABILITIES image shows a bivariate normal distribution applied at every positive pixel in the SELECT image to aid in final assessment of spot locations, and NUMBERS contains number codes at pixel locations for spot identification and size quantification. This algorithm seems to perform well in simulated gels, but faces difficulties with regard to diffuse or saturated spots. Further, this algorithm does not account for spot matching and, thus, cannot be used for comparative analysis across gels; see [43] for details.

Miecznikowski et al. [44] apply a (hyper-)crossical-shaped structuring element (i.e., shaped like a multidimensional cross) of varying size to an image (creating a “*Smooth*” image), and use the smoothing decomposition, *Data = Smooth + Rough* to determine the associated residual (i.e., “*Rough*”) image. This structuring element with arm-size *c* is combined with a median operation over the pixels within the cross shaped window. When the median operation is applied to the preprocessed image (as described in [45]), the associated residual image contains crosses whose centers are the local maxima. Focusing our attention on the rough image, [46] isolates the positive intensities and applies mathematical morphology (erosion and dilation) to remove the noise and heighten the presence of the crosses. The nonlinear nature of the median operator allows this method to detect proteins of low intensity as well as nearby proteins within a gel. Thus, with this method we have a means to identify spot centers and estimate spot sizes; see [44, 46] for details.

Other spot detection procedures include modeling 2D-Gaussians, applying diffusion equations, linear programming, and wavelet modeling as described in [47]. The use of 2D-Gaussians, however, is disputed due to the knowledge that spots can be oddly shaped, and thus cannot be accurately represented via a 2D-Gaussian model. Ultimately, it is difficult to obtain the algorithm details for many of the proprietary softwares that are marketed in the industry. This severely limits the ability to understand exactly the feature detection methods employed to locate and quantify spots in a gel image.

## 6. Discussion

Experiments utilizing the described proteomic platforms have the general goal of deriving knowledge of the biological system. Through the experiments utilizing these platforms, the formal hypotheses are tested on the basis of the experimental data. Common hypotheses to examine include differential expression, cluster analysis, association with a phenotype, and correlation with survival (or other censored variables). There are standard statistical methods designed to handle each of these situations; for example, see [48] for details. With all of these platforms, consideration should be given to the issue of multiple testing. In proteomic experiments, multiple testing can arise when (1) examining thousands of peptides for differential expression, (2) testing a peptide against several different contrasts, or (3) examining the significance of groups of peptides for association with a given phenotype. In these situations, scientists need to choose a Type I error rate and a method to control it. Guidance for these choices is provided in [49, 50]. The statistical aspects and the assumptions underlying the choice of error rate and control method are often critical to the success of proteomic experiments.

Before we can tackle such high-level analyses, we must first have sufficiently and satisfactorily determined the appropriate data summary information to address these problems. This matter, however, has not been addressed so that a uniform procedure is established and accepted. This demonstrates the significance of low-level analysis! Biological and medical communities have not uniformly accepted a low-level analysis procedure for preprocessing proteomics data and thus have many available methods from which to choose for performing low-level analysis of such data structures. Proteomic technologies are not based on the hybridization of complementary DNA strands, hence it is not possible to engineer quality control experiments for proteomic data as it is for microarrays. Further, sample preparation, starting materials, and reagents and differences in MS machines and gel imagers have contributed to the wide variability in the data; for example, the sensitivity of the techniques to specimen collection and handling is an issue [51]. Similar to the situation with preprocessing algorithms, inconsistent sample preparation and handling can lead to spurious results and conclusions. Further confounding the problem is that many of the methods to analyze MS and gel data are proprietary, and thus not fully disclosed, while the field lacks a suitable “gold standard” to fully evaluate the available methods. These aspects of analyzing proteomic data are difficult to simulate and thus there is a need for a comprehensive set of experiments that can accurately assess each aspect of the data analysis pipeline in gel-based and non-gel-based experiments.

None of these procedures are fully automated as they generally require additional user input to determine thresholding parameters or local window ranges for consideration. Further, these input parameters can influence each stage in the sequence of preprocessing steps. Algorithm results are generally inconsistent and unrecoverable, which causes great concern on its impact on the determination of scientifically significant proteins. As noted in [52] regarding mass spectrometry data, different preprocessing algorithms could severely affect downstream analyses, and so the choice of procedure must be approached carefully; the same is true for two-dimensional gel electrophoresis data, non-gel-based data or, more generally, any image or spectral data.

Another challenge for scientists that further complicates the field's ability to establish a generally-accepted preprocessing approach is its ability to detect “small” proteins, that is, proteins that are present in low abundance but are differentially expressed or modified. The high-abundance proteins with large peaks are generally uninteresting as they are already extensively studied for their ability to serve as biomarkers. Currently, researchers are searching for the more elusive, low-abundance proteins. For example, in cancer biomarker research, studies commonly attempt to quantify proteins or metabolites that are shed into the blood stream by the tumor (see, e.g., [53, 54]). These proteins are present in relatively low abundance, and thus are represented in mass spectra by small or low-lying peaks, and analogously in gel data as low-lying or faint spots; however, they represent a promising set of biomarkers in cancer diagnosis. Many such peaks are usually undetected because of the signal-to-noise ratio, leading to larger false negatives. For example, in ICAT studies, noisy data hinder low-lying peak detection, and thus the identification of associated peak pairs [34]. This is a significant problem for scientists since it limits their ability to detect and evaluate potential biomarkers and peptides.

So, which approach is “best” for preprocessing proteomic data according to the respective methodology? Model accuracy, false discovery rates, the ability to detect low abundance protein peaks, and the user intervention required of the procedures are significant factors that play a role in addressing this question. These significant and substantial factors influencing preprocessing techniques for proteomic data therefore make this question difficult (if not impossible) to answer without a large, cohesive effort across the proteomics and statistics communities. Only through such an endeavor can we truly make significant forward movement toward a generally accepted approach for data analysis.

## Figures and Tables

**Table 1:** Comparison table of gel-based and non-gel-based methods for proteomic data analysis. This table highlights some benefits and drawbacks to many popular technological approaches in analyzing protein samples. GB and NGB, respectively, denote the associated technique as gel-based or non-gel-based.

Analysis Method	Benefits	Drawbacks
2-DE-MS and DIGE-MS (GB)	1. DIGE minimizes gel-to-gel variation	1. low-abundant protein identification
	2. DIGE produces better spot matching	2. sensitive to experimental and technological variation
	3. allows for study of protein change on large scale	3. laborious process
	4. strong resolving power	4. difficulties automating procedure
	5. high sensitivity	5. protein comigration
	6. low equipment cost	6. study replication may be prohibitive

LC-MS and LC-MS/MS (GB)	1. fast procedure	1. difficulty analyzing low-abundance proteins
	2. easily automated high resolution measurements	2. not quantitative
		3. Expensive machines
	3. MS/MS improves the detection limits for some compounds	4. MS/MS spectrum TIC decreased compared to MS spectrum TIC
	4. MS/MS improves S/N ratio relative to MS	5. Ion activation methods affect spectra efficiency, reproducibility, feature detection

ICAT (NGB)	1. accurate relative quantification	1. missed identification of proteins containing little to no cysteine residue (i.e., cysteine-content biased)
	2. reduces peptide mixture complexity	
	3. compatible with various fractionation methods	2. posttranslational modifications missed
	4. at least as sensitive as DIGE	3. complex interpretation of MS/MS spectra when biotin group added
		4. noise impacts peak detection for ICAT peaks with low expression levels
		5. compounds may dilute through LC column at different speeds

iTRAQ (NGB)	1. greater sensitivity than DIGE and ICAT	1. occasional inherent problem due to timed-ion selector resolution of tandem mass spectrometer
	2. can perform relative or absolute quantification in four phenotypes	2. compounds may dilute through LC column at different speeds

MudPIT (NGB)	1. orthogonality of the chromatographic phases in the separation process	1. does not allow for identification of the site at which probe labeling occurs
	2. robust representation of separated proteins peptides, from complex peptides	2. since the proteins are broken down to their component, any information about modifications and isoforms is lost.
		3. large computing power required to complete database searching
		4. the approach is generally limited to use with organisms that have complete genome sequence data available for searching

## References

[1] Serra J (1982). *Image Analysis and Mathematical Morphology*.

[2] Zhuang X, Haralick RM (1986). Morphological structuring element decomposition. *Computer Vision, Graphics, and Image Processing*.

[3] Soille P (2003). *Morphological Image Analysis: Principles and Applications*.

[4] Maragos P (1987). Tutorial on advances in morphological image processing and analysis. *Optical Engineering*.

[5] Gavrila DM, Giebel J, Perception M, Res DC, Ulm G Shape-based pedestrian detection and tracking.

[6] Tarassenko L, Hayton P, Cerneaz N, Brady M Novelty detection for the identification of masses in mammograms.

[7] Saber E, Tekalp AM (1998). Frontal-view face detection and facial feature extraction using color, shape and symmetry based cost functions. *Pattern Recognition Letters*.

[8] Wang Y, Chua C-S, Ho Y-K (2002). Facial feature detection and face recognition from 2D and 3D images. *Pattern Recognition Letters*.

[9] Schena M, Shalon D, Davis RW, Brown PO (1995). Quantitative monitoring of gene expression patterns with a complementary DNA microarray. *Science*.

[10] Shalon D, Smith SJ, Brown PO (1996). A DNA microarray system for analyzing complex DNA samples using two-color fluorescent probe hybridization. *Genome Research*.

[11] Monteoliva L, Albar JP (2004). Differential proteomics: an overview of gel and non-gel based approaches. *Briefings in Functional Genomics and Proteomics*.

[12] Domon B, Aebersold R (2006). Challenges and opportunities in proteomics data analysis. *Molecular and Cellular Proteomics*.

[13] O’Farrell PH (1975). High resolution two dimensional electrophoresis of proteins. *The Journal of Biological Chemistry*.

[14] Ünlü M, Morgan ME, Minden JS (1997). Difference gel electrophoresis: a single gel method for detecting changes in protein extracts. *Electrophoresis*.

[15] Levner I (2005). Feature selection and nearest centroid classification for protein mass spectrometry. *BMC Bioinformatics*.

[16] JEOL Mass Spectrometers (2006). *Tandem Mass Spectrometry (MS/MS)*.

[17] de Hoffmann E (1996). Tandem mass spectrometry: a primer. *Journal of Mass Spectrometry*.

[18] Delahunty CM, Yates JR (2007). MudPIT: multidimensional protein identification technology. *BioTechniques*.

[19] Gygi SP, Rist B, Gerber SA, Turecek F, Gelb MH, Aebersold R (1999). Quantitative analysis of complex protein mixtures using isotope-coded affinity tags. *Nature Biotechnology*.

[20] Wu WW, Wang G, Baek SJ, Shen RF (2006). Comparative study of three proteomic quantitative methods, DIGE, cICAT, and iTRAQ, using 2D gel- or LC-MALDI TOF/TOF. *Journal of Proteome Research*.

[21] Gan CS, Chong PK, Pham TK, Wright PC (2007). Technical, experimental, and biological variations in isobaric tags for relative and absolute quantitation (iTRAQ). *Journal of Proteome Research*.

[22] Meleth S, Deshane J, Kim H (2005). The case for well-conducted experiments to validate statistical protocols for 2D gels: different pre-processing = different lists of significant proteins. *BMC Biotechnology*.

[23] Kang Y, Techanukul T, Mantalaris A, Nagy JM (2009). Comparison of three commercially available DIGE analysis software packages: minimal user intervention in gel-based proteomics. *Journal of Proteome Research*.

[24] Rosengren AT, Salmi JM, Aittokallio T (2003). Comparison of PDQuest and Progenesis software packages in the analysis of two-dimensional electrophoresis gels. *Proteomics*.

[25] Raman B, Cheung A, Marten MR (2002). Quantitative comparison and evaluation of two commercially available, two-dimensional electrophoresis image analysis software packages, Z3 and Melanie. *Electrophoresis*.

[26] Chang J, Van Remmen H, Ward WF, Regnier FE, Richardson A, Cornell J (2004). Processing of data generated by 2-dimensional gel electrophoresis for statistical analysis: missing data, normalization, and statistics. *Journal of Proteome Research*.

[27] Marengo E, Robotti E, Antonucci F, Cecconi D, Campostrini N, Righetti PG (2005). Numerical approaches for quantitative analysis of two-dimensional maps: a review of commercial software and home-made systems. *Proteomics*.

[28] R Development Core Team (2008). *R: A Language and Environment for Statistical Computing*.

[29] Gentleman RC, Carey VJ, Bates DM (2004). Bioconductor: open software development for computational biology and bioinformatics. *Genome Biology*.

[30] Du P, Kibbe WA, Lin SM (2006). Improved peak detection in mass spectrum by incorporating continuous wavelet transform-based pattern matching. *Bioinformatics*.

[31] Smith CA, Tautenhahn R xcms: LC/MS and GC/MS Data Analysis.

[32] Robinson M *Flagme: Fragment-Level Analysis of GCMS-Based Metabolomics Data*.

[33] Cuadros-Inostroza A, Redestig H, Hannah MA, Potsdam G The TargetSearch Package.

[34] Yu W, Qi RZ, Liu J, Zhao H Mass spectrometry based quantitative proteomics data analysis methods: a review.

[35] Yasui Y, Pepe M, Thompson ML (2003). A data-analytic strategy for protein biomarker discovery: profiling of high-dimensional proteomic data for cancer detection. *Biostatistics*.

[36] Fushiki T, Fujisawa H, Eguchi S (2006). Identification of biomarkers from mass spectrometry data using a “common” peak approach. *BMC Bioinformatics*.

[37] Coombes KR, Fritsche HA, Clarke C (2003). Quality control and peak finding for proteomics data collected from nipple aspirate fluid by surface-enhanced laser desorption and ionization. *Clinical Chemistry*.

[38] Coombes KR, Tsavachidis S, Morris JS, Baggerly KA, Hung M-C, Kuerer HM (2005). Improved peak detection and quantification of mass spectrometry data acquired from surface-enhanced laser desorption and ionization by denoising spectra with the undecimated discrete wavelet transform. *Proteomics*.

[39] Morris JS, Coombes KR, Koomen J, Baggerly KA, Kobayashi R (2005). Feature extraction and quantification for mass spectrometry in biomedical applications using the mean spectrum. *Bioinformatics*.

[40] Hoopmann MR, Finney GL, MacCoss MJ (2007). High-speed data reduction, feature detection, and MS/MS spectrum quality assessment of shotgun proteomics data sets using high-resolution mass spectrometry. *Analytical Chemistry*.

[41] von Haller PD, Yi E, Donohoe S (2003). The application of new software tools to quantitative protein profiling via isotope-coded affinity tag (ICAT) and tandem mass spectrometry II. Evaluation of tandem mass spectrometry methodologies for large-scale protein analysis, and
the application of statistical tools for data analysis and interpretation. *Molecular and Cellular Proteomics*.

[42] Srinark T, Kambhamettu C (2008). An image analysis suite for spot detection and spot matching in two-dimensional electrophoresis gels. *Electrophoresis*.

[43] Langella O, Zivy M (2008). A method based on bead flows for spot detection on 2-D gel images. *Proteomics*.

[44] Miecznikowski JC, Sellers KF, Eddy WF (2009). *Multidimensional Median Filters for Finding Bumps*.

[45] Sellers KF, Miecznikowski J, Viswanathan S, Minden JS, Eddy WF (2007). Lights, camera, action: systematic variation in 2-D difference gel electrophoresis images. *Electrophoresis*.

[46] Miecznikowski JC (2006). *Spot Detection in Two-Dimensional Electrophoresis Images*.

[47] Soggiu A, Marullo O, Roncada P, Capobianco E (2009). Empowering spot detection in 2DE images by wavelet denoising. *In Silico Biology*.

[48] Gentleman R, Carey VJ, Huber W, Dudoit S, Irizarry RA (2005). *Bioinformatics and Computational Biology Solutions Using R and Bioconductor*.

[49] Miller RG (1981). *Simultaneous Statistical Inference*.

[50] Dudoit S, Van Der Laan MJ (2008). *Multiple Testing Procedures with Applications to Genomics*.

[51] Rai AJ, Gelfand CA, Haywood BC (2005). HUPO Plasma Proteome Project specimen collection and handling: towards the standardization of parameters for plasma proteome samples. *Proteomics*.

[52] Baggerly KA, Morris JS, Coombes KR (2004). Reproducibility of SELDI-TOF protein patterns in serum: comparing datasets from different experiments. *Bioinformatics*.

[53] Semmes OJ, Feng Z, Adam B-L (2005). Evaluation of serum protein profiling by surface-enhanced laser desorption/ionization time-of-flight mass spectrometry for the detection of prostate cancer: I. Assessment of platform reproducibility. *Clinical Chemistry*.

[54] Yu KH, Rustgi AK, Blair IA (2005). Characterization of proteins in human pancreatic cancer serum using differential gel electrophoresis and tandem mass spectrometry. *Journal of Proteome Research*.

